# Modifiable Parent Factors Associated with Child and Adolescent School Refusal: A Systematic Review

**DOI:** 10.1007/s10578-022-01358-z

**Published:** 2022-04-10

**Authors:** Meena Chockalingam, Kayan Skinner, Glenn Melvin, Marie B. H. Yap

**Affiliations:** 1 Turner Institute for Brain and Mental Health, School of Psychological Sciences, Monash University, Melbourne, Australia; 2 School of Psychology, Centre for Social and Early Emotional Development, Deakin University, Melbourne, Australia; 3 Melbourne School of Population and Global Health, University of Melbourne, Melbourne, Australia

**Keywords:** School refusal, Modifiable parent factors, Children, Adolescents, Anxiety

## Abstract

School refusal is a complex problem that refers to difficulty attending/remaining at school due to emotional distress about attendance. Despite its occurrence being associated with negative outcomes, many are unresponsive to the current treatment options. While parent factors have a key role to play in school refusal, they are not adequately addressed in existing treatments. Further research is needed to consolidate understanding and implement new treatments. Employing the PRISMA method, this review aims to identify modifiable parent factors associated with child and/or adolescent school refusal. Eight studies met inclusion criteria from which nine factors were identified. Factors found to be associated with school refusal included: parent psychopathology, family functioning and maternal overprotection (communication subdomain). Other factors such as maternal overprotection (affection, assistance and travel subdomains) and parental self-efficacy had weak or inconsistent results warranting further investigation. Overall, findings call for action in this field that has sparse and dated literature.

## Introduction

In most countries, school attendance is mandatory until a specified age. While a certain level of fear or anxiety about school attendance is normal, 1–2% of school-aged children [1, 2] experience distressing and dysfunctional levels of this fear and anxiety. Termed school refusal, this condition refers to difficulties attending and/or remaining at school due to emotional distress. Berg [3] identified five defining criteria for school refusal, comprising: (a) reluctance or refusal to attend school; (b) being at home during school hours with parental knowledge; (c) emotional distress at the prospect of school; (d) an absence of severe antisocial behaviours beyond resistance to parental attempts to enforce school attendance; and (e) reasonable yet ineffective attempts by parents to enforce school attendance. School refusal is not a psychiatric diagnosis in the Diagnostic and Statistical Manual-5 [4], however individuals with school refusal commonly have primary diagnoses of anxiety- and/or mood-related disorders [5–7]. Though variable, common reasons why children refuse school include difficulty separating from parents, wanting to avoid aversive stimuli associated with school (e.g. the bus ride to school) and/or having a specific or generalized anxiety related to school (e.g. fear of being asked to talk in front of the class) [5, 8]. School refusal presentations are heterogeneous and can include tantrums on school mornings, refusing to leave the house for school, leaving school when arousal increases, or remaining at school but staying out of the classroom (e.g. in the sick bay or library).

School refusal is one condition under the umbrella term of ‘school attendance problems’, which refers to any problematic absence from school for explained or unexplained reasons [9]. School refusal is distinguished from other types of attendance problems, most notably school withdrawal, where parents actively encourage nonattendance [10] and truancy, where children stay out of school and attempt to conceal this fact from their parents [7–10]. This review will focus solely on school refusal, known in earlier research as school phobia.

Evidence to-date about the development, maintenance and treatment of school refusal suggest that interventions can be targeted at three levels: (1) the individual (child/adolescent), (2) the school, and (3) the family. While intervention is likely needed at all three levels, the role of the parents or carers (henceforth ‘parents’) within the family is the focus of this study for several reasons. First, parents are inherently involved in school refusal as per Berg’s [3] definition, yet their best efforts have not been successful. Second, parents can play an integrating role between the child and school and third, a child is influenced by and depends on parent behaviours to overcome their distress and school refusal experience. In addition, parents can be an effective and perhaps even more accessible target than the child for an intervention to support school-refusing children [11].

### Detrimental nature of School Refusal

School refusal detrimentally affects young people and their families in the short and long term. In the short term, school-refusing young people may face breakdowns in their family/peer relationships, increased family conflict, social withdrawal, declines in their academic performance, high emotional distress on a daily basis [6], and co-occurring mental illness [5, 12]. The relationship between school refusal and mental illness is likely bidirectional, however the presence of school refusal can lead to or exacerbate mental illness. Possible mechanisms for this exacerbation include social isolation, family conflict, lack of academic progress and avoidance of anxiety provoking stimuli at school e.g. interacting with peers. Significant long-term consequences of school refusal include reduced prospective career opportunities, poorer interpersonal relationships, disrupted social, emotional and intellectual development [3, 13], and higher rates of lifetime mental disorders [14, 15]. Furthermore, the duration of school refusal predicts future outcomes; with longer periods being associated with lower probability of successful school re-engagement [16] and more distressing and detrimental consequences [6]. Most cases of school refusal do not remit without treatment [17]. In addition, parents/caregivers also experience significant distress in attempting to manage the issue [18] and therefore may have reduced capacity to facilitate school re-engagement [19].

#### Need for targeted intervention for School Refusal

The distressing and detrimental path of this condition highlights the need for strong evidence-based interventions. Cognitive Behavioural Therapy (CBT) is currently suggested to be the most efficacious treatment for school refusal [20]. However, when examining CBT studies for the treatment of school refusal, it becomes evident that there is still a substantial proportion of school-refusing children that do not respond to CBT. In the Last et al. [21] study, post-treatment school attendance rates were 67% for children who received CBT for school refusal, meaning children were in the classroom 67% of the time in the past school week. While 65% of children (*n* = 13) who received CBT for school refusal reached 95% attendance rates, a seemingly good response, there was no significant group X time difference when compared to improvements in the control group. This means there is no evidence that CBT for school refusal was superior to the control group which received educational-support therapy. Similarly, in the Melvin et al. [22] study, though attendance rates increased post-intervention (for adolescents who received CBT with or without medication), post-treatment attendance rates (52%) were still below the target of 80%. This indicates that further exploration is needed into the mechanisms underlying school refusal and how it can best be treated.

#### Parent involvement in interventions for School Refusal

Existing school refusal interventions have not been seen to improve attendance rates to required levels. Studies suggest that school refusal is very difficult to treat without some form of parental involvement [23, 24]. Some CBT studies have involved parents by conducting individual parent sessions [22, 25–27] or involving them as part of the child sessions [21, 28]. Both these modalities primarily focus on teaching the parents what the child is being taught. Heyne, King [26] evaluated the role of parent and teacher involvement in augmenting child focused CBT for school refusal. All treatment groups (child therapy only, parent and teacher training only and both child therapy and parent/teacher training) demonstrated statistically and clinically significant improvements in school attendance between pre- and post-treatment time points. This indicates that a treatment approach that did not involve the child directly, versus child-only treatment, or combined can be equally as effective. This finding has been mirrored amongst children with anxiety disorders where a treatment approach targeting parents solely produced a non-inferior effect to a child-targeted CBT intervention [29].

Parents play a critical role in the prevention and treatment of mental health problems in children and adolescents [30]. While not all school-refusing children suffer from a diagnosed mental illness, the high co-occurrence indicates that it is beneficial to understand the well-established literature linking child mental health and parenting. Studies exploring child and adolescent mental health identified a myriad of modifiable parent factors that can increase or decrease risk of anxiety and depression disorders such as over-involvement, warmth and autonomy granting [31, 32]. Furthermore, it has also been established that parenting programs can improve outcomes for child and adolescent mental health by targeting relevant parenting behaviours, skills, self-efficacy and barriers to effective parenting (e.g. parental psychopathology) [33–35].

Overall, there is a need for more effective treatment of school refusal. Though existing programs involve parents, they do not adequately address and target parent-level factors as proposed in Sandler et al.’s [35] framework. This framework suggests that to produce long-term effects in parenting and youth outcomes, parents must be actively involved in using parenting skills, building parental self-efficacy and reducing barriers to effective parenting [35]. These parenting changes produce effective long-term effects by influencing parents’ own and their child’s social, cognitive, biological and behavioural processes [35]. It is possible that targeting more of these factors will help augment existing treatments (i.e. targeted at the child) or be effective treatment approaches in themselves (i.e. without involving the child). To date, there has been limited research and consolidated understanding into which parent-level factors are specifically related to school refusal in children and adolescents.

### Next Steps and Focus of Review

Current interventions for school refusal, as described above, primarily involve parents by teaching them what their child is being taught. Unlike targeted parenting interventions, such interventions may not adequately target the modifiable parental factors implicated in school refusal. Parenting interventions have been shown to improve not only parenting factors, but also have shown meaningful change in child/adolescent behavioural and emotional problems [35–37]. In order to inform parenting interventions to prevent or address school refusal, we need to understand what modifiable parent factors are associated specifically with school refusal. Extending from the definition of Yap and colleagues [32, 38, 39], modifiable parent factors refer to those within parents’ capacity to alter or influence themselves (i.e. parenting behaviours, communication and family functioning) or those they can access support to alter (e.g. current parental psychopathology). This contrasts with parent factors that are difficult or impossible to change at the familial or individual level (i.e. ethnicity, socio-economic status and family history of psychopathology). To date, there has been no systematic review of the literature to amalgamate the current understanding of parent factors associated with school refusal. The aim of this systematic literature review is to identify what modifiable parent factors have been associated with child and/or adolescent school refusal.

## Method

### Search Strategy

Studies were identified using a systematic search of five electronic databases: Embase, OVID Medline, PsycINFO, Scopus and Education Resource Information Centre (ERIC). The combination of systematic search terms was designed according to the Preferred Reporting Items for Systematic Reviews and Meta-Analyses (PRISMA) statement [40] and comprised of text words and index terms (MeSH) relating to parenting, child/adolescence and school refusal. Terms were chosen based on prior research and key word searching. The original search was conducted 2nd May 2019 and an updated search was conducted 11th June 2020. No restriction on publication date was imposed. Additional sources were identified by hand searching reference lists of included studies. The search strategy, inclusion and exclusion criteria and data-analysis methods were pre-specified, prospectively registered and published on the PROSPERO database (ID CRD42019134508).

#### Study selection

Studies were included if they met the following criteria: (a) Published in the English language; (b) Published in a peer reviewed journal; (c) Child participants are in primary or secondary school and are experiencing school refusal (as defined by Berg et al., [3]); (d) Measured at least one modifiable parent factor. Studies were excluded if: (a) No full text was available; (b) Study did not evaluate a school refusal group against comparable non-school refusal sample, or in a treatment study, there was no continuous measure of school attendance; (c) Parent factor was not measured with a validated measure; (d) Children were not in primary or secondary school age-range or school-attending children were not sub-analysed; (e) A composite measure of school refusal was used and individual components were not separately analysed (e.g. a measure combining school refusal and truancy, and analyses did not separate between participants experiencing school refusal and those who were truant); (f) A composite measure of parenting was used and individual components were not separately analysed.

#### School Refusal Definition

Berg’s [3] definition of school refusal was utilised in this review to identify school refusal samples and exclude studies with samples of school refusal behaviour which includes both school refusal and truancy. Though we intended to only include studies that strictly adhered to Berg’s [3] criteria, most studies did not discuss or meet all criteria. Hence relevant papers were classified into three bands based on their fit to Berg’s [3] criteria (refer to Table 1). Band 1 included those that addressed and met all five criteria. Studies categorised in Band 2 met at least the two core criteria: a) reluctance or refusal to attend school and c) emotional distress at the prospect of school. The most commonly missing and/or unmet criterion in this band was e) reasonable yet ineffective attempts by parents to enforce school attendance. Studies in Band 3 did not meet core criteria and were hence excluded from the review. Of the included studies, two were in Band 1 and six were in Band 2. An imperfect fit was tolerated because of the meagre nature of the literature in this area and that the majority of relevant studies fell within this category.

#### Screening and data extraction

Using Covidence systematic review software v.1471 [41], one researcher (M.C or K.S) screened titles and abstracts of all studies identified through the electronic database search once duplicates were removed, with 10% of studies cross-checked. Full text articles were then independently reviewed by two authors (M.C and K.S). Discrepancies were resolved by discussion between authors and in consultation with senior authors (G.M and M.Y). All reasons for exclusion were documented.

A data-extraction spreadsheet was created and pre-piloted on 5 studies. Two researchers (M.C and K.S) independently extracted study characteristics and outcomes. Study characteristics included: country of participant population, demographic information about child and parent samples, sample size and sample type (clinical or community). Predictor variable (parent/family factor) details extracted included: factor name, factor description, measure utilised, informant and target (i.e. mother, father, family). The outcome of interest (school refusal) details extracted included: duration of school refusal, how the study defined school refusal, method of assessment for school refusal, and inclusion/exclusion criteria. Furthermore, *p* values for the association between parent factor/s and school refusal were also extracted for each included study. Authors of eligible papers that did not report data required for this review were contacted in an attempt to gain necessary information.

#### Categorisation of parent factors

Parent factors were identified from each included study and reviewed by authors (M.C, G.M and M.Y) based on the name, description and items in the measure. Factors that were comparable were grouped together and appropriately labelled based on the construct they targeted. Given the scantiness of the literature, factors measured in one or more eligible studies were included. As shown in Table 1, nine factors were identified: overall parent psychopathology, parent depressive symptoms, parent anxiety symptoms, family functioning, parental self-efficacy, and the affection, communication, assistance and travel subdomains of maternal overprotection.

**Table 1:** Categorisation and measurement of parent factors

*Parenting Factor (predictor)*	*Definitions*	*Measures*	*Sample Items*	*Response Scale*
Overall parental psychopathology	Broad range of psychological symptoms and psychiatric morbidity	SCL-90-R^a^ SCID-I^b^	“For the past week, how much were you bothered by headaches?” “How did you react when (trauma) happened? (were you very afraid or did you feel terrified or helpless?)”	5-point frequency scale: Not at all – Extremely. Clinician rated: Absent or false; subthreshold; threshold or true
Parent depressive symptoms	Symptoms of depression currently present	BDI^c^ BDI-II^d^	“**Sadness** (0) I do not feel sad; (1) I feel sad; (2) I am sad all the time and can’t snap out of it; (3) I am so sad and unhappy and I can’t stand it” “**Pessimism** (0) I am not discouraged about my future; (1) I feel more discouraged about my future than I used to; (2) I do not expect things to work out for me; (3) I feel my future is hopeless and will only get worse”	
Parent anxiety symptoms	Symptoms of anxiety currently present	STAI^e^ BAI^f^	“I am tense” “Indicate how much you have been bothered by numbness or tingling?”	4-point frequency scale: Almost never – Almost always. 4-point scale: Not at all – Severely, it bothered me a lot.
Family functioning	Overall social and structural properties of a family environment including interactions (levels of conflict and cohesion), adaptability, organisation and quality of communication	FAM^g^ FAD-GF^h^ FES^i^	“We spend too much time arguing about what our problems are” “Planning family activities is difficult because we misunderstand each other” “Family members really help and support one another”	4-point scale: Strongly agree – Strongly disagree. 4-point scale: Strongly agree – Strongly disagree. True; False
Maternal overprotection – Affection	How much the mother prefers and encourages affection from their child	SADQ^j^ – Preference Scores (rate based on preference NOT actual)	“Does he/she [child] come close to you for affectionate contact (e.g. sitting on knee or putting arm around, *do not* include kissing)?”	5-point frequency scale: Less than once a week or not at all – More than once a day (several times a day)
Maternal overprotection - Communication	How much the mother prefers and encourages communication from their child	SADQ^j^ – Preference Scores (rate based on preference NOT actual)	“Did he/she talk things over with you and ask your help about what was going on at school? (exclude homework)”	5-point frequency scale: Less than once a week or not at all – More than once a day (several times a day)
Maternal overprotection - Assistance	How much the mother prefers and encourages their child to ask for assistance with daily tasks	SADQ^j^ – Preference Scores (rate based on preference NOT actual)	“Did you wash or bath him/her (not including hair washing?)”	5-point frequency scale: Less than once a week or not at all – More than once a day (several times a day)
Maternal overprotection - Travel	How much the mother prefers and encourages their child to travel away from home	SADQ^j^ – Preference Scores (rate based on preference NOT actual)	“Did he/she go on a bus without you?”	5-point frequency scale: Less than once a week or not at all – More than once a day (several times a day)
Parental self-efficacy	An individual’s appraisal of his/her competence in the parental role	SEQ-RSAP^k^ Parenting Sense of Competency Scale – Efficacy Subscale	“If my child has difficulty attending school, I know what can be done to address this.” “Being a parent is manageable, and any problems are easily solved.”	4-point scale: Totally disagree – Totally agree. 6-point scale: Strongly agree – Strongly disagree.

^a^Symptom Checklist-90-Revised.

^b^Structured Clinical Interview for the DSM-IV Axis I Disorders.

^c^Beck Depression Inventory.

^d^Beck Depression Inventory-II.

^e^State-Trait Anxiety Inventory for Adults.

^f^Beck Anxiety Inventory.

^g^Family Assessment Measure.

^h^Family Assessment Device – General Functioning Scale.

^i^Family Environment Scale.

^j^Self-administered Dependency Questionnaire.

^k^Self-Efficacy Questionnaire for Responding to School Attendance Problems.

#### Risk of Bias

The risk of biases for each study was assessed independently by two authors (M.C. and K.S.) using the checklist derived by Hayden et al. [42]. Discrepancies were resolved through discussion. The tool included six domains: (1) Study participation, (2) Study attrition, (3) Prognostic factor measurement, (4) Outcome measurement, (5) Study confounding and (6) Statistical analysis and reporting, of which 5 domains were deemed relevant and therefore utilised (refer to Table 2). The tool provided prompting items and considerations which were taken together to inform the judgement of each bias domain. Each domain received an overall rating of low, moderate or high risk of bias with some being marked as non-applicable (NA) if relevant.

**Table 2:** Summary of Risk of Bias Tool [42]

Domain	Summary	Prompting Items
Study Participation	The study sample adequately represents the population of interest	a. Adequate participation in the study by eligible persons
		b. Description of the source population or population of interest
		c. Description of the baseline study sample
		d. Adequate description of the sampling frame and recruitment
		e. Adequate description of the period and place of recruitment
		f. Adequate description of inclusion and exclusion criteria
Study Attrition	The study data available (i.e., participants not lost to follow-up) adequately represent the study sample	a. Adequate response rate for study participants
		b. Description of attempts to collect information on participants who dropped out
		c. Reasons for loss to follow-up are provided
		d. Adequate description of participants lost to follow-up
		e. There are no important differences between participants who completed the study and those who did not
Prognostic Factor (PF) Measurement	The PF is measured in a similar way for all participants	a. A clear definition or description of the PF is provided
		b. Method of PF measurement is adequately valid and reliable
		c. Continuous variables are reported or appropriate cut points are used
		d. The method and setting of measurement of PF is the same for all study participants
		e. Adequate proportion of the study sample has complete data for the PF
		f. Appropriate methods of imputation are used for missing PF data
Outcome Measurement	The outcome of interest is measured in a similar way for all participants	a. A clear definition of the outcome is provided
		b. Method of outcome measurement used is adequately valid and reliable
		c. The method and setting of outcome measurement is the same for all study participants
Statistical Analysis and Reporting	The statistical analysis is appropriate, and all primary outcomes are reported	a. Sufficient presentation of data to assess the adequacy of the analytic strategy
		b. Strategy for model building is appropriate and is based on a conceptual framework or model
		c. The selected statistical model is adequate for the design of the study
		d. There is no selective reporting of results

### Data Analysis

If studies included multiple measures assessing a particular parent factor, an electronic randomiser was utilised to include only one relevant measure per association of interest. A meta-analysis could not be conducted due to the high level of methodological heterogeneity between the included studies, including the way associations were analysed and reported, the way the predictor (parent) and outcome (school refusal) factors were measured, and the study samples. The weighted *z* score test was selected as an appropriate method to combine *p* values to determine whether associations between parent factors and child and adolescent school refusal were reliable. This method has been found to be more precise and have greater power than non-weighted methods like Fisher-*Z* [43]. The analysis involved calculating a weight for each study (w_*i*_ = *n* -3) and multiplying this by *Z* (the standardised *z* scores from each extracted association). The weighted *z* score will equal the sum of this product (Z × w_*i*_) divided by the square root of the sum of w_*i*_-squared (w_*i*_ ^2). When the resultant weighted *Z* score corresponded to a *p* value lower than 0.05, the null hypothesis was rejected. If an included study reported a significant result without the exact *p* value (e.g., *p* < 0.01), and the given information was not sufficient for estimating the *p* value, then the cut-off score was used (e.g. 0.05, 0.01, 0.001, etc.). Furthermore, if a non-significant result was reported without an exact *p* value, and the given information was not sufficient for estimating the *p* value, the value of 0.5 was used. Alongside quantitative analyses, results are also presented in a narrative synthesis.

## Results

**Fig. 1:**
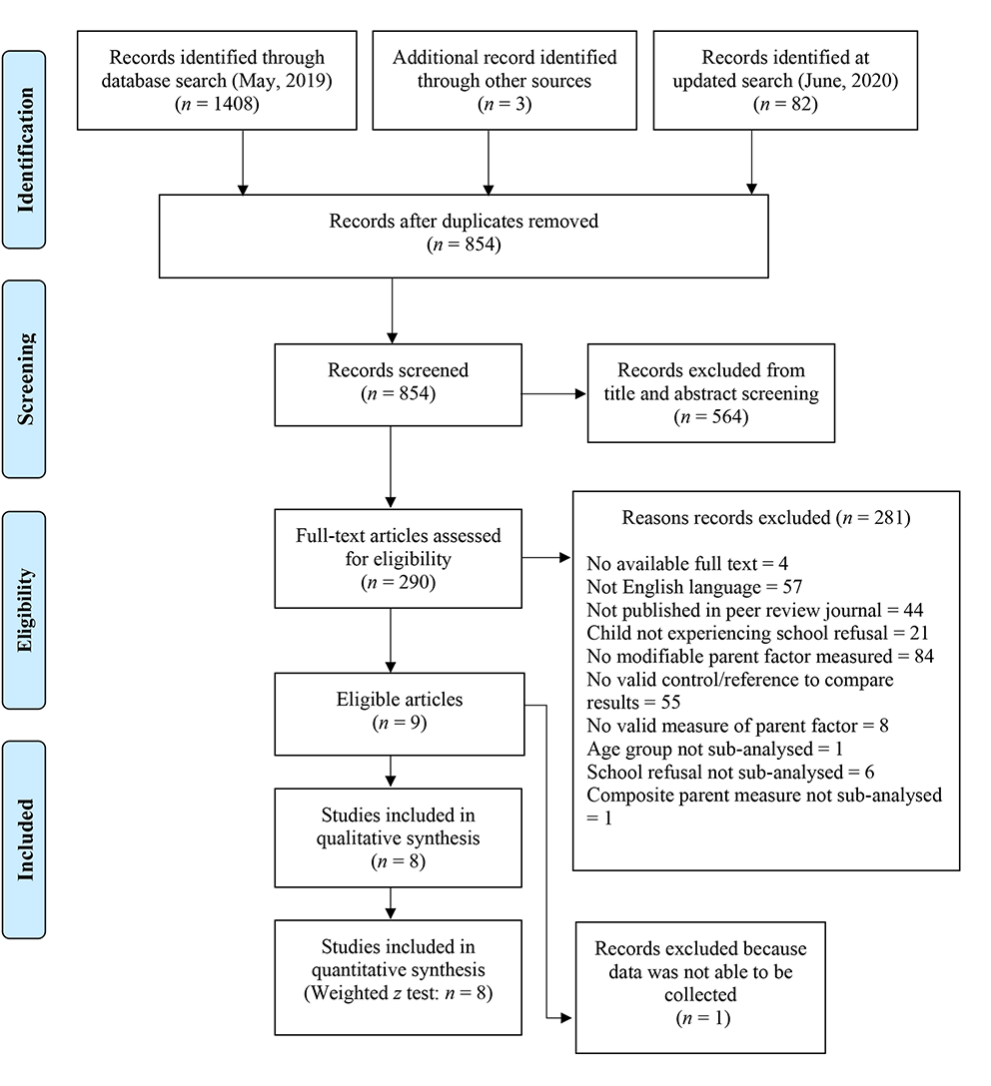
PRISMA flowchart

Figure 1 illustrates the systematic literature search process following Preferred Reporting Items for Systematic Reviews and Meta-Analyses (PRISMA) guidelines [40]. A total of 1493 papers were retrieved through various search methods. After the removal of duplicates, 854 titles and abstracts were screened, of which 290 papers were then assessed for eligibility based on full-text (see Fig. 1). The 8 included studies were published between 1974 and 2015 and consisted of 7 cross-sectional case control studies and 1 treatment study. Across the 8 studies, 725 children/adolescents (mean age = 13.3) and 907 parents participated. Refer to Table 3 below for a description of other sample characteristics. A quality assessment was conducted and results summarised in Table 4. Ratings indicate study quality varied. Furthermore, a summary of the included study characteristics are presented in Table 5.

**Table 3:** Sample characteristics

Characteristics		No. of Studies
Country	Australia	1
	England	1
	Netherlands	1
	Turkey	2
	USA	3
Child Age Range	Primary School (> 4–12 years)	1
	Secondary School (> 11–19 years)	3
	Mixed	3
	Unknown	1
Child Gender	< 50% Male	3
	> 50% Male	3
	Unknown	2
Parent Gender	< 50% Male	5
	> 50% Male	0
	50% − 50%	3
School Refusal Group Recruited From	In-patient clinic	1
	Out-patient clinic	5
	Community	1
	Unknown	1
Comparison Group Recruited From	In-patient clinic	0
	Out-patient clinic	1
	Community	6
	Unknown	0
	Not Applicable	1

**Table 4:** Summary of risk of bias for included studies

Studies	Study participation	Study attrition	Prognostic factor Measurement	Outcome Measurement	Statistical Analysis and Reporting
Bahali et al. [44]	Moderate	n/a	Low	Moderate	Low
Berg and McGuire [45]	High	n/a	Moderate	High	Low
Bernstein and Garfinkel [46]	Moderate	n/a	Low	Moderate	Low
Carless et al. [47]	Low	n/a	Low	Low	Low
Hansen et al. [48]	Low	n/a	Low	Low	Low
Heyne et al. [27]	Low	Moderate	Low	Low	Low
Last and Strauss [49]	Low	n/a	Low	Moderate	Low
Ozcan et al. [50]	Moderate	n/a	Low	Moderate	Low

**Table 5:** Summary of included study characteristics

Studies	Child Participants	Parent Participants	School Refusal Definition Banding	Parent Factors Examined	Measure/s	Main findings
Bahali et al. [44]	Over the age of 5 School-refusing (clinical sample), *n* = 55^a^ Control (community sample), *n* = 56^a^	Parents of school-refusers, *n* = 110 Parents of controls, *n* = 112	Band 2	Overall Psychopathology Depressive Symptoms Anxiety Symptoms	Symptom Checklist-90 Revised Beck Depression Inventory State-Trait Anxiety Inventory	*p* < 0.02 Mothers, *p* < 0.0001 Fathers, *p* < 0.0003 Mothers, *p* < 0.0001 Fathers, *p* < 0.0001
Berg and McGuire [45]	Secondary school – no age range reported School-phobic (clinical sample), *n* = 39 Non-school-phobic (other psychiatric cases), *n* = 58 Controls (community sample), *n* = 128	Parents of school-phobics, *n* = 39^a^ Parents of non-school-phobics, *n* = 58^a^ Parents of controls, *n* = 128	Band 2	Maternal Overprotection	Self-administered Dependency Questionnaire – Preference scores	Affection subscale, *p* < 0.05 Communication subscale, *p* < 0.001 Assistance subscale, *p* > 0.05 Travel subscale, *p* > 0.05
Bernstein and Garfinkel [46]	Aged between 7–18 School phobia (clinical sample), *n* = 6 Other disorders (clinical sample), *n* = 5	Parents of school phobics, *n* = 12 Parents of other disorders, *n* = 10	Band 2	Family Functioning Parent psychopathology but no data reported	Family Assessment Measure – third revision	*p* < 0.04
Carless et al. [47]	Aged between 12–17 School-refusing (clinical sample), *n* = 60 School-attending (community sample), *n* = 46	Parents of school refusers, *n* = 60 Parents of school attenders, *n* = 46	Band 1	Depressive symptoms Anxiety symptoms Parental self-efficacy Family Functioning	Beck Depression Inventory – II State-Trait Anxiety Inventory Parenting sense of competence scale – efficacy subscale Family Assessment Device – General Functioning Scale	p < 0.01 *p* < 0.01 *p* < 0.01 *p* < 0.01
Hansen et al. [48]	Aged between 6–17 School-refusing (clinical sample), *n* = 76	Parents of school refusers, *n* = 76^a^	Band 2	Family Functioning	Family Environment Scale	*p* < 0.001
Heyne et al. [27]	Aged between 11–17 School-refusing adolescent, *n* = 20	Parents of school refusers, *n* = 32	Band 1	Parental self-efficacy	Self-efficacy Questionnaire for Responding to School Attendance Problems	Mothers, *p* = 0.153 Fathers, *p* = 0.636
Last and Strauss [49]	Aged between 7–17 School-refusing (clinical sample), *n* = 63 Control (community sample matched for age and sex, never psychiatrically ill), *n* = 63	Parents of school refusers, *n* = 63^a^ (only 54 completed SADQ) Parents of controls, *n* = 63^a^	Band 2	Maternal Overprotection	Self-administered Dependency Questionnaire – Preference scores	Affection subscale, N/R (non-significant) Communication, N/R (non-significant) Assistance, *p* = 0.04 Travel, N/R (non-significant)
Ozcan et al. [50]	Aged between 6–12 School phobia (clinical sample), *n* = 25 Control (community sample – matched for age and sex, free of any psychiatric diagnosis), *n* = 25	Parents of school phobics, *n* = 50 Parents of controls, *n* = 50	Band 2	Overall Psychopathology Depressive Symptoms Anxiety Symptoms Social Anxiety	Structured Clinical Interview for DSM-I Beck Depression Inventory Beck Anxiety Inventory Liebowitz Social Anxiety Scale	Mothers, *p* = 0.002 Fathers, *p* = 0.017 *p* < 0.001 *p* < 0.001 *p* < 0.001

^a^ *Exact number not reported in paper, estimate calculated based on information provided*

Note. Band 1 studies included those that addressed and met all five of Berg’s [3] school refusal criteria. Band 2 studies met at least Berg’s [3] two core criteria; a) reluctance or refusal to attend school and c) emotional distress at the prospect of school

Note. *p* values are shown as they were reported by the authors

### Parent psychopathology

#### Overall parent psychopathology

Three associations from two independent studies [50, 44] were included in the weighted *z* test which yielded a significant result (*P* = 0.001), indicating that parents of children with school refusal had higher rates of psychological problems (i.e. Symptom Checklist-90-Revised) and/or psychiatric morbidity (i.e. Structured Clinical Interview for the DSM-IV Axis I Disorders) than parents of children without school refusal. Both samples had 50–50% representation of mothers and fathers.

#### Parent depressive symptoms

Based on five associations across three studies [47, 50, 44], school refusal was related to higher levels of parent depressive symptoms (combined *P* < 0.001). Two of the three studies collected self-report symptom data from an equal number of mothers and fathers, and found significant associations for both genders. In these two studies, mothers and fathers of children (aged across primary and secondary schooling) with school refusal reported more depressive symptoms than mothers and fathers of children without school refusal [50, 44]. Average symptom scores for both parent-gender groups were within the non-clinical range. The third study mirrored these results but did not examine mothers and father separately [47].

#### Parent anxiety symptoms

Five associations were extracted from three studies [47, 50, 44] and yielded a significant result in the weighted *z* test (*P* < 0.001). The results indicated that parents of children with school refusal had higher anxiety levels compared to parents of children without school refusal. This finding held true for both mothers and fathers in the two studies that conducted separate analyses for mothers and fathers [50, 44]. Across the three studies, average symptom severity was in the clinical range for parents of children with school refusal, in contrast to the parents with children without school refusal, where average symptom severity was in the non-clinical range. This was consistent across the studies except for one [50], which found that fathers with school-refusing children had scores in the non-clinical range, though the score was significantly higher than the fathers without school-refusing children.

#### Family Functioning

Though four studies were eligible, relevant data from one study was not retrievable despite attempts to contact the authors, therefore this study was excluded [51]. The consequent weighted *z* test included three associations from three independent studies [46–48]. A significant combined *P* value was found (*P* < 0.001) indicating that family functioning, as reported by parents, was significantly lower for families with children who are refusing school when compared to families with children without school refusal.

#### Maternal overprotection

Maternal overprotection was measured in two studies [45, 49] using preference scores from the self-administered dependency questionnaire (SADQ) as reported by mothers. The preference scores were collected from four domains – affection, communication, assistance and travel. Two associations were extracted per domain and included in each weighted *z* test. There was no difference between parents with and without school-refusing children on affection (*P* = 0.072), assistance (*P* > 0.5) and travel (*P* > 0.5) domains of overprotection. However, the communication subscale yielded a significant association (*P* < 0.001), denoting that mothers of children with school refusal prefer and encourage more communication than mothers of children without school refusal.

#### Parental self-efficacy

Two associations from two independent studies [27, 47] were included in a weighted *z* test yielding a significant result (*P* = 0.007). The studies differed in their design; one cross-sectional and one treatment study comparing parental self-efficacy across pre- and post-treatment for adolescents with school refusal. Results on the treatment study [27] indicate that the cross-sectional association between school attendance in adolescents (reported as percentage of time spent at school during 10 school days preceding assessment) and parental self-efficacy at the pre-treatment time point was non-significant. In contrast, Carless et al.’s [47] cross-sectional study found significantly lower levels of parental self-efficacy in parents of school-refusing adolescents, compared to parents of school-attending adolescents. The overall weighted-*z* score encompassing both *p* values indicated a significant negative association between parental self-efficacy and school refusal. Further interrogation of this finding is complicated by the difference in methodology between the studies. The studies do stand as comparable as both relied on similar criteria for school refusal with school attendance variable in the Heyne et al. [27] being a measure of school refusal severity.

## Discussion

This systematic review identified 8 studies that examined 9 associations between various parent factors and school refusal in children and adolescents. Based on the studies, five parent factors (overall parental psychopathology, depressive symptoms, anxiety symptoms, family functioning and the communication subdomain of maternal overprotection) were found to have a reliable association with school refusal, in the expected direction. Four factors (affection, assistance and travel subdomains of maternal overprotection and parental self-efficacy) had weak or inconsistent associations with school refusal.

The risk of bias assessment revealed the variable quality of studies in this field. The mix of low, moderate and high ratings could reflect the dated nature of multiple papers as research quality has evolved. Furthermore, the results under the domain ‘outcome measure’ expose the inconsistent quality and manner in which school refusal has been measured in children and adolescents. Finally, the overall quality of the studies to answer our research question is low, with 7 of the 8 included studies having a cross-sectional design.

Parent factors will be discussed in two sections – factors with emerging evidence and factors with weak or inconsistent evidence. Within the emerging factors, three of the five factors with reliable associations, fit within the broader domain of parent mental health and will therefore be discussed together. Within the four weak/inconsistent factors, three will be discussed together because of the broader association with maternal overprotection. Broader themes of paternal involvement, limitations and clinical implications will then be explored.

### Factors with an emerging evidence base

#### Parental Mental Health

The current review revealed an emerging evidence base for parents of school-refusing children and adolescents having a higher prevalence of poor mental health, compared to parents of school-attending children and adolescents. Across three studies, this association was found when parental mental health was measured as overall psychopathology, depressive symptoms, and anxiety symptoms.

Using different measures, two studies demonstrated an association between overall parent psychopathology and school refusal. The robustness of this association is affirmed by the findings for parental depression and anxiety symptoms. A combination of the Beck Depression Inventory and Beck Depression Inventory II was used in the three studies to measure parent depressive symptomatology. Although depressive symptom levels were higher across all studies for parents with school-refusing children in comparison to parents without school-refusing children, mean scores were in the non-clinical range. This suggests that even within the non-clinical range, higher levels of parent depressive symptoms are associated with school refusal. This association is potentially bi-directional. These parents may not have diagnosable depressive disorders, but they may still experience increased depressive symptoms because school refusal is so challenging for parents to manage. Conversely, children of parents with depressive symptoms may be more likely to develop school refusal. Though school refusal is considered an anxiety-based condition, its occurrence can be precipitated by symptoms of depression including low motivation, low self-worth and lack of energy [6]. Given established relationships between parent mood disorders and increased risk of psychiatric problems in children [1, 28], children of parents with increased depressive symptoms may have a higher likelihood of developing school refusal.

Similarly, anxiety symptomatology was higher across the three studies, for parents with school-refusing children in comparison to parents without school-refusing children. However, studies varied in the severity of these symptoms for parents with school-refusing children. Bahali, Tahiroglu [44] and Carless, Melvin [47] found that for both mothers and fathers, mean anxiety scores as measured by the State-Trait Anxiety Inventory were in the moderate/high range (i.e. clinical range) for parents with school-refusing children in comparison to in the no/low anxiety range (i.e. non-clinical range) for parents with school-attending children. Ozcan, Kilic [50] found a similar finding for mothers using the Beck Anxiety Inventory but found that although fathers with school-refusing children had significantly increased anxiety symptoms in comparison to fathers without school-refusing children, scores for fathers with school-refusing children were in the mild range (i.e. non-clinical range). Clinically, this suggests that anxiety plays a more significant role in the mental health of parents with school-refusing children than parents without school-refusing children. The inconsistent result for fathers may reflect the lower rates of depression and anxiety reported by males [52].

As found in this review, school refusal studies have consistently reported co-occurrence of psychopathology within the family, specifically anxiety disorders [53]. Given the majority of papers assessed in this review utilised a correlational study design, it is not possible to ascertain the direction of the relationship. The finding could reflect a transmission of mental health difficulties from parent to child. With the high prevalence of mental illness in the child and adolescent school refusal population, this finding could highlight a genetic propensity [54] to mental illness in particular anxiety-based conditions. As school refusal is an anxiety-based condition, the finding that parents (mothers and sometimes fathers) more commonly had anxiety symptoms in the clinical range may reflect a predisposition for the children to experience anxiety and therefore develop school refusal. The finding may also highlight how psychopathology might transmit environmentally within a family system. Drawing from Bandura’s social learning theory, parental modelling of anxiety has been explored and supported in research [55]. The development of school refusal may be aided by the transmission of anxious thinking and behaviour from parent/s to child [56]. In addition, parents with anxiety are more likely to engage in family accommodation of child anxiety with school attendance (e.g. parents directly or indirectly facilitating the avoidance of school to alleviate their child’s distress) [57]. This could hinder their ability to support school re-engagement and instead reinforce anxious behaviours in their child, which maintains school refusal. Furthermore, the child can miss opportunities to develop adaptive coping and emotional regulation skills for managing their anxiety.

In contrast, these findings could represent a transmission of symptoms from child to parent. With school refusal being a challenging condition for parents to manage, the struggles experienced may initiate parental mental health difficulties, stress, exhaustion, low mood, frustration and parental/family conflict [18]. This impact may be a similar phenomenon to ‘carer burden’ in carers of people with mental illness [58].

With consistent significant results across the studies using the most rigorous definition of school refusal (Band 1 (*n* = 1); Band 2 (*n* = 2)), findings appear to be true to a school refusal presentation. Overall, though research can further explore the direction of this relationship, the evidence to date paints a strong narrative for the relationship between parent mental health and child school refusal.

##### Family Functioning

The review’s findings support prior theoretical understanding of the family being involved in the aetiology of school refusal [51, 59, 60]. Across three cross-sectional studies, parents of children with school refusal reported poorer family functioning than parents of children without school refusal. The construct of family functioning can be sub-analysed using multiple domains. Though the three included studies use different measures, each with their own set of domains, three key overlapping domains were identified: Role performance (organisation, clarity and fairness of tasks assigned to family members and whether these tasks are completed responsibly), control (manner in which family expresses, implements and maintains standards of behaviour) and involvement (quality of interest and concern for wellbeing between family members and to the extent to which they help/support). Further insight into the relationship of these domains and school refusal is not available in the scarce literature. Given high prevalence of anxiety disorders in school refusal presentation, the child and adolescent anxiety literature may provide relevant insights. This literature show similar significant negative associations between child anxiety and family functioning [53, 61, 62]. However specific domains have mixed findings. In Jongerden and Bögels’ [63] study where families with anxiety disordered children and adolescents were compared with convenience sample control families, family relational functioning (encompassing involvement domain) was found to be significantly different, however family control (encompassing control and role performance domains) was not significant different between anxiety-disordered and control families. Further research can explore whether control and role performance domains may be specifically associated with school-refusal rather than the broader anxiety category.

Furthermore, Hansen and colleagues [48] found that families with school-refusing children were more likely to come from homes that place relatively low emphasis on recreational activities such as seeing family/friends and going out for entertainment. A reduction in these activities is one common form of family accommodation of child anxiety [57] and perpetuating factor of child depression [64], and therefore may play a role in the maintenance of school refusal. Additionally, this finding aligned with the anxiety literature where Jongerden and Bögels [63] also found a significant difference in active-recreational activity between families with and without anxiety-disordered children.

Though the above literature has been identified, directionality of the association between family functioning and school refusal is unknown. While a bi-directional relationship likely exists, the extent to which poor family functioning precedes and/or maintains school refusal is yet to be explored. In spite of directionality, further exploration and inclusion of relevant family functioning subdomains within school refusal intervention appears relevant.

##### Maternal overprotection – communication

Overprotective parenting has been implicated in child anxiety disorders [56, 64]. Rapee’s [65] and Wood et al. [66] reviews both present sufficient evidence to support a reliable relationship between parental over control and child anxiety. It is suggested that overprotective behaviours convey the message that the world is dangerous, reinforce avoidance, inhibit child psychological autonomy and limit children’s opportunities to develop skills and confidence to tackle challenges [67]. Given the high prevalence of anxiety disorders in school-refusing children, this review’s finding mirrors and extends previous findings in providing support for the role of maternal protection in school refusal.

Results from two cross-sectional studies indicate that mothers with school-refusing children prefer and encourage increased communication in comparison to mothers without school-refusing children. This finding is consistent with the broader literature. The maternal overprotection – communication subscale from the Self-Administered Dependency Questionnaire (SADQ), measures the extent to which a mother prefers and encourages their child to communicate with them about their lives in topics including school, friends, homework. Preferring high rates of communication may inhibit a child’s psychological autonomy, decrease their self-confidence and in turn reinforce the anxious and/or depressive thinking and behaviours that maintain their poor school attendance [53]. Overprotective parents may ask too many questions (portraying a message of distrust), expect children to talk through all decisions with them, and overly shape a child’s thinking. Age-appropriate autonomy granting is important across child developmental stages however it is particularly pertinent during adolescence, when increasing autonomy is a necessity for reaching developmental milestone [68]. This over-protective behaviour may then be particularly detrimental for adolescents and may partly account for the greater severity of school refusal during this stage of development, including its increased resistance to treatment [24].

A number of mechanisms could be underlying this significant result. Firstly, mothers’ own anxiety could contribute to her encouraging over-dependency. This anxiety may interfere with the parent’s adaptive coping skills which in turn may mean a parent exhibits more ‘anxiety-enhancing’ parenting behaviours [69]. Given the significant associations found between parent anxiety and school refusal, the increased levels of parental anxiety may contribute to these overprotective parenting behaviours. Secondly, another contributing factor could be mothers not granting age-appropriate autonomy [70]. Thirdly, parents may have a lack of belief in their child’s ability to cope. Guzell and Vernon-Feagans [71] found that parents who have a lower sense of control over difficult care-giving situations were more likely to use over-controlling parenting strategies. Overall, parenting behaviour may play a significant part in the development and maintenance of school refusal and therefore could be beneficial to address in subsequent treatment.

### Factors with a Weak/Inconsistent evidence base

#### Maternal overprotection – affection, travel and assistance

Findings from this review found no significant differences in maternal overprotection in the domains of affection, travel and assistance, between mothers of children with and without school refusal. This is inconsistent with general literature discussed above suggesting that overprotective behaviours play a role in the development and maintenance of child anxiety disorders. In the domain of affection, the two included studies were inconsistent in their findings. These inconsistencies suggest that further research is needed to explore the role of mothers’ encouraging affection in school refusal.

Furthermore, the two included studies consistently found no association between overprotective behaviours in the domains of assistance and travel, and school refusal. This finding is paralleled in Last and Strauss’ [49] study where though children with school refusal asked for more assistance from their mothers than control children, no evidence was found that mothers prefer this behaviour. These findings may indicate that parenting behaviours surrounding assistance and travel domains are not significantly associated with school refusal.

However, several limitations of the SADQ measure used in both included studies need to be considered when interpreting the findings regarding maternal overprotection. First, the SADQ questions were not targeted for age of the child. Mothers with children ranging between 7 and 17 years old were answering questions such as ‘Did you wash or bath him/her (not including hair washing)’ and ‘Did he/she come into your bed for company at night or in the early morning’. Both studies included in this review had a mean child age that fell in the adolescent age range meaning that parents in both groups may be answering similarly primarily because the questions were not relevant to their child. Second, the SADQ does not directly measure overprotection. Overprotection in newer measures utilise more contemporary definitions that refer to parenting behaviours that restrict a child’s exposure to situations which the parents may perceive as potentially threatening to the child. Utilising preference scores from the SADQ may indirectly measure this, however more direct and recently validated measures (e.g., Parental Overprotection Scale by Clark et al. [67]) should be explored as well. Lastly, the parent-reported measures are not objective. Some studies exploring parental overprotection utilise independent ratings of observed parent-child interactions to gain a more objective measure [72]. This limitation is not specific to the SADQ as many parenting behaviour measures are self-reports. Though independent ratings of parenting behaviour are considered the gold standard in the field of parenting research, they come with their own limitations in terms of participant burden, cost and hence feasibility.

##### Parental self-efficacy

Despite conflicting results in the two studies analysed, results from the weighted *z* score analysis indicate that parental self-efficacy and school refusal were found to have a significant negative association in adolescents. Carless et al. [47] cross sectional study found that parents of school-refusing adolescents had lower levels of parental self-efficacy than parents of school-attending adolescents. They suggested that for the school-refusing population, perceived parenting failures in managing their child’s distress may lead to lower perseverance with enforcing school attendance. This failure to support their child’s re-engagement may in turn confirm their low parental self-efficacy creating a bidirectional relationship between child outcomes and parental self-efficacy. Heyne et al.’s, [27] findings extended this idea using a non-randomised trial of developmentally sensitive cognitive behavioural therapy. In contrast, they found that parental self-efficacy was not significantly associated with school attendance rates. The inconsistent results may be accounted for by methodological differences between studies: Carless et al., [47] compared parental self-efficacy in parents with school-refusing adolescents to parents of school-attending adolescents; whereas Heyne et al.’s [27] association was derived from the correlation between parental self-efficacy and child school attendance (proxy for school refusal severity) as a continuous variable at pre-treatment. Furthermore, the inconsistencies may be explained by the vast differences in how parental self-efficacy is measured in the literature [73, 74] with the Carless et al., [47] using the efficacy subscale of the parenting sense of competence scale and Heyne et al., [27] using the Self-Efficacy Questionnaire for Responding to School Attendance Problems. In addition, with the scarce number of studies identified in this review, findings are not conclusive and therefore further research is recommended. The influence of parental factors such as self-efficacy may need to be explored in more detail across the range of presentations and severity levels seen in school refusal. The small number of studies in this field precluded such sub-analyses in the current review.

##### Other parent factors

This review highlights the sparse and dated (no published study between 2015 and 2020) nature of the school refusal and parenting literature. Though nine modifiable parent factors were identified, several potential factors were not able to be included because the studies did not meet selection criteria. Some theoretically driven factors that can be the focus of future research include family factors such as adaptability (family ability to change its structure, role relationships and rules to respond to needs), cohesion (emotional bonding between family members), communication (verbal and non-verbal information exchanges between family members), role performance (allocation, willingness and enactment of roles), affective expression, family control (process by which family members influence one another) and relationship dynamics [75–77]. Furthermore, there may be other relevant factors that have not yet been explored highlighting the need for more research in this field.

### Paternal involvement in School Refusal

There is a limited understanding of the role of fathers, as fathers are often under-represented in parenting and family research [78]. The current review supports previous findings that paternal mental health is related to child psychopathology [79]. Significant associations were identified between depression, anxiety and overall psychopathology symptomology in fathers and child school refusal across studies. Though these findings are consistent for mothers, research suggests that mothers and fathers have unique contributions to child functioning [79] therefore further research assessing both parents separately is warranted. Findings from this review reinforce calls for further research to explore maternal and paternal roles in child and adolescent school refusal presentations. Research in this field is of increasing importance as the responsibility for managing school refusal often falls onto the mother without much considerations of the father’s potential role. Exploring paternal role can help involve fathers in a beneficial manner, share responsibility and augment child outcomes.

### Limitations

The results of this review should be interpreted in light of its limitations. First, as discussed, the sparse nature of the school refusal literature limited the depth and breadth of parental factors that could be examined in this review. Further studies are needed to be able to draw sound conclusions on the role of identified parent factors in school refusal. Additionally, the limited number of studies restricted sub-analyses. The severity and presentation of school refusal can vary substantially between children and adolescents, so further research is needed to examine developmental differences in the role of parent factors. Furthermore, given the evolving definition of school refusal, several older studies were excluded in the screening stages due to not meeting our inclusion criteria which required school refusal definitions in Band 1 or 2 of Berg’s [3] criteria. In addition, the majority of the included studies were cross-sectional meaning it was not possible to ascertain causality or explore the direction of the relationships between the parent factors and school refusal.

Statistically, a meta-analysis was not able to be conducted to quantitatively synthesise the findings. Instead, the weighted *z* score method of combining *p* values was utilised as it can be applied to studies that analyse data in a variety of ways [43].

### Clinical implications and conclusions

This review identified and synthesised parent factors that have been examined and associated with school refusal presentations across children and adolescents. Consistent with findings from Wei and Kendell’s [80] systematic review, this review suggests that incorporation of parent-targeted treatment components can be effective for complex presentations of child anxiety such as school refusal. Across the factors identified in the scant literature, parent mental health as a broad construct would seem to be the most promising target for intervention. Though the direction of the relationship is still uncertain, emerging evidence was identified linking current parent mental health to the presence of school refusal in children. Further treatment of school refusal could explore whether supporting parent mental health can indirectly improve the child’s school-refusal presentation. Targeting parent mental health may also be an effective form of prevention. In addition, factors with promising evidence include family functioning, maternal overprotection – communication and factors with weak/inconsistent evidence include parental self-efficacy, maternal overprotection – affection, assistance and travel and child dependency. All these factors need further research to better understand their relationships with school refusal.

The review provides a starting point in identifying potential modifiable parental factors that can be used to inform parent-targeted school refusal interventions. This review’s findings validate the existence of promising modifiable parent factors, however emphasise the need for substantially more research in this field. Furthermore, examining whether directly targeting these parent factors in interventions can improve child outcomes would pave a way forward in enhancing current interventions for school refusal.

## Summary

Overall, this review aimed to collate existing literature and identify modifiable parent factors that are associated with child and adolescent school refusal. School refusal is defined as difficulty attending/remaining at school due to emotional distress about attendance that occurs despite parental attempts to enforce attendance. This review is important and relevant to conduct as existing treatment options for school refusal (which largely focuses on child-targeted strategies and techniques) have been found to only work for some, leaving a substantial proportion of school-refusing children without an efficacious treatment option. As parents play a key role in both the development and maintenance of school refusal, having a better understanding of the parent’s role could lead to more parent-targeted strategies or techniques used to enhance interventions. Using the Preferred Reporting Items for Systematic Reviews and Meta-Analyses (PRISMA) method, this review identified eight studies that met inclusion criteria, from which nine modifiable parent factors were identified and presented in quantitative analysis and narrative synthesis. Factors found to be reliably associated with school refusal included parent psychopathology, family functioning and maternal overprotection (communication subdomain). The other identified factors, maternal overprotection - affection, assistance and travel subdomains and parental self-efficacy, had weak or inconsistent results that warrant further investigation. Key limitations include the sparse and dated nature of the literature and hence authors acknowledge that identified associations are preliminary and that there may be other factors that are relevant but have not yet been empirically studied and hence are not noted in this review. Findings provide a base for future research in this area but also speak loudly in rallying for more action in this field.
